# Astragaloside IV Protects against Shear Stress-Induced Glycocalyx Damage and Alleviates Abdominal Aortic Aneurysm by Regulating miR-17-3p/Syndecan-1

**DOI:** 10.1155/2024/2348336

**Published:** 2024-02-13

**Authors:** Guojian Li, Qionghui Yang, Kaikai Luo, Ankou Xu, Lijuan Hou, Zhaoxiang Li, Lingjuan Du

**Affiliations:** ^1^Department of Vascular Surgery, Affiliated Hospital of Yunnan University, Kunming, Yunnan, China; ^2^Department of Pharmaceutical Sciences, The Third People's Hospital of Yunnan Province, Kunming, China; ^3^Department of Vascular Medicine, People's Hospital of Hekou Yao Autonomous County, Kunming, China

## Abstract

**Background:**

The present study aimed to analyze the impact of astragaloside IV (AS-IV) on abdominal aortic aneurysm (AAA) and the glycocalyx, elucidating the potential mechanism of AS-IV.

**Methods:**

Rat models of AAA were established using porcine pancreatic elastase. The effects of intraperitoneal AS-IV injection on the morphology, diameter, and glycocalyx of the aorta and the expression of miR-17-3p and Syndecan-1 (SDC1) protein were examined. Differentially expressed miRNAs from peripheral blood samples of healthy individuals, untreated patients with AAA, and treated patients with AAA were identified through sequencing. The relationship between miR-17-3p and SDC1 was validated using a dual-luciferase reporter assay. In vitro, shear stress was induced in human aortic endothelial cells (HAECs) to simulate AAA. Overexpression of miR-17-3p was performed to assess the effects of AS-IV on miR-17-3p and SDC1 expressions, apoptosis, and glycocalyx in HAECs.

**Results:**

AS-IV mitigated aortic damage in AAA rats, reducing the aortic diameter and alleviating glycocalyx damage. In addition, it suppressed the increase in miR-17-3p expression and promoted SDC1 expression in AAA rats. Peripheral blood miR-17-3p levels were significantly higher in patients with AAA than in healthy individuals. miR-17-3p inhibited the SDC1 protein expression in HAECs. In the in vitro AAA environment, miR-17-3p was upregulated and SDC1 was downregulated in HAECs. AS-IV inhibited miR-17-3p expression, promoted SDC1 expression, and mitigated shear stress-induced apoptosis and glycocalyx damage in HAECs. Overexpression of miR-17-3p blocked AS-IV–induced SDC1 expression promotion, glycocalyx protection, and apoptosis suppression in HAECs.

**Conclusion:**

miR-17-3p may damage the glycocalyx of aortic endothelial cells by targeting SDC1. AS-IV may promote SDC1 expression by inhibiting miR-17-3p, thereby protecting the glycocalyx and alleviating AAA.

## 1. Introduction

Abdominal aortic aneurysm (AAA) is characterized by a tumor-like expansion of the abdominal aorta, marked by a diameter increase exceeding 50% [1]. Prevalent among elderly men, particularly smokers, the male-to-female ratio stands at 10 : 3 [2, 3]. Globally, AAA afflicts 2%–3% of the population, escalating to approximately 8.8% in individuals over 65 years [4, 5]. With a staggering 94% mortality rate upon rupture, current clinical interventions primarily rely on surgery and risk factor reduction [6–8]. Consequently, understanding the molecular mechanisms driving AAA development is crucial for formulating effective treatment strategies.

The degradation of the glycocalyx, a dynamic barrier between the blood vessel wall and blood, plays a pivotal role in shear stress-induced damage to aortic endothelial cells, a key pathological feature of AAA [9–12]. Syndecan-1 (SDC1), a core protein anchoring the glycocalyx to aortic endothelial cells through a transmembrane domain, is integral to its structural integrity [13]. SDC1 damage results in glycocalyx shedding, a phenomenon implicated in various vascular diseases, including inflammation and atherosclerosis [14–16]. Given the significance of inflammation and atherosclerosis in AAA development [17] and the demonstration of shear stress-induced glycocalyx damage in sepsis [18], it is imperative to explore the relationship between shear stress and glycocalyx damage in AAA.

Astragaloside IV (AS-IV), a small-molecule saponin (molecular weight = 784) derived from *Astragalus*, is noted for its anti-inflammatory properties [19, 20]. Recognized for its protective effects on the vascular system, AS-IV has been shown to inhibit hypoxia–induced pulmonary arterial remodeling [21] and mitigate vascular endothelial cell damage induced by high glucose levels [22, 23]. While previous studies have demonstrated the ability of AS-IV to alleviate AAA by regulating macrophages and suppressing inflammation [24], its impact on the glycocalyx and aortic endothelial cells in AAA remains unexplored.

In the posttranscriptional regulatory mechanism, miRNAs such as miR-24 [25] and miR-143-5p [26] have been implicated in AAA. Notably, miR-17-3p, associated with the interaction of vascular endothelial cells and inflammatory cells, has been linked to AAA formation [27]. A previous study suggested that mesenchymal stem cell exosomes can mitigate AAA inflammation by delivering miR-17 to target NLRP3 [28]. The elevated miR-17-3p expression has been linked to shear stress, a significant AAA induction mechanism [29, 30]. However, the precise role of miR-17-3p on AAA and its impact on the glycocalyx remains unclear.

The present study aimed to investigate the impact of AS-IV on AAA and elucidate its mechanism of action, focusing on the shedding of the glycocalyx in aortic endothelial cells. The findings promise novel insights into AAA treatment strategies.

## 2. Materials and Methods

### 2.1. Construction of Rat Models of AAA and AS-IV Treatment

A total of 40 Sprague–Dawley rats (Certificate number: SCXK(京)-2019-0010; weight: 180–220 g; Shanghai Slack Experimental Animal Center, China) were divided into four groups (*n* = 10): Sham, AAA, AAA + AS-IV-40, and AAA + AS-IV-80 groups. Rats in the AAA group underwent anesthetization with 1.5% isoflurane inhalation, maintaining a body temperature of 37°C with a heating pad. Following an abdominal incision to expose the aorta, a polyethylene catheter (Braintree Scientific, Braintree, MA, USA) was inserted, and 12 U/mL porcine pancreatic elastase (PPE; Sigma-Aldrich, MO, USA) was gradually injected over 30 min. Aortic diameter was assessed 14 days postinjection, achieving modeling success rates of 100%, 90%, 80%, and 90%, respectively, for the four groups. Rats in the Sham group received an equivalent volume of high-temperature-inactivated PPE (heated at 90°C for 45 min). Rats in the AAA + AS-IV-40 and AAA + AS-IV-80 received 40 and 80 mg/kg AS-IV, respectively, via gavage once daily for 14 consecutive days, commencing on the first day after PPE injection [20, 31]. The survival rates were 100%, 70%, 70%, and 80%, respectively, for the four groups. All experiments were performed in accordance with the Affiliated Hospital of Yunnan University animal experimental guidelines and approved by the Animal Ethics Committee of the Affiliated Hospital of Yunnan University Animal (NO. YNU20220148).

### 2.2. Imaging

On the 14^th^ postoperative day, rats were anesthetized with 1.5% isoflurane inhalation, and ultrasonography (Zonare, Mountain View, CA, USA) was used to measure the maximum aortic diameter. The ultrasonography probe operated at a frequency of 12 MHz.

### 2.3. Hematoxylin and Eosin (H&E) Staining

Rats were euthanized through inhalation of excess carbon dioxide, and abdominal aortic tissues were collected. After fixation in 10% neutral formalin, the tissues were sectioned, deparaffinized, and subjected to hematoxylin staining at 25°C for 10 min followed by eosin staining at 25°C for 3 min. Stained tissue sections were observed using an inverted microscope (K-FRAME-60, KOSTER, China).

### 2.4. Transmission Electron Microscopy

Abdominal aortic tissues were fixed overnight at 4°C in 2.5% glutaraldehyde and 1% osmic acid for 2 hr. Following fixation, tissue samples were washed with phosphate-buffered saline (PBS), embedded in paraffin, and subjected to observation of glycocalyx morphological characteristics on the inner wall of the aortic vessel using a transmission electron microscope (GSS-600, SHSIWI, China).

### 2.5. Immunohistochemical (IHC) Staining

Fixed tissue samples underwent treatment with 0.01% Triton X-100 for 10 min, followed by PBS washing. Subsequently, samples were exposed to 0.03% hydrogen peroxide for 10 min and washed twice with PBS for 3 min each. Normal serum was added dropwise, and samples were incubated in a wet box at 37°C for 15 min to eliminate nonspecific staining. After discarding the normal serum, anti-SDC1 antibody (1 : 500, ab128936) was added dropwise, and samples were incubated in a wet box at 37°C for 1 hr and then at 4°C overnight. The next day, tissue samples were incubated with the secondary antibody in a wet box at 37°C for 15 min and washed twice with PBS for 3 min each. DAB staining was performed using a kit with A, B, and C reagents mixed in 1 mL of distilled water each. The stained samples were incubated at 25°C and observed under a microscope to control the reaction time. For counterstaining, samples were immersed in hematoxylin for 2 min, washed under tap water, differentiated with a hydrochloric acid–alcohol solution, stained blue with ammonia solution, and washed under tap water. Subsequently, samples were dehydrated, cleared, and observed under a microscope (DMIRB, Leica).

### 2.6. miRNA Sequencing and Differential Expression Analysis

miRNA sequencing and subsequent differential expression analysis were conducted following the procedures outlined in our previous study [32].

### 2.7. RNA Fluorescence In Situ Hybridization (FISH)

FISH was used to assess the expression of miR-17-3p in the abdominal aortic tissues of rats. Tissues were fixed in 4% paraformaldehyde at 25°C for 1 hr, permeabilized with an 800 *μ*L permeabilization solution for 15 min, and underwent two washes with PBS for 5 min each. Following this, tissue samples were rinsed once with PBT–RNA hybridization solution (1 : 1) and then stored in the RNA hybridization solution. The RNA hybridization solution was boiled for 5 min and quenched on ice for 5 min. Subsequently, samples were added to the hybridization solution and incubated at 56°C for 2 hr. Probes, diluted with the RNA hybridization solution (1 : 500), were heated at 80°C for 5 min and then quenched on ice for 5 min. The samples were combined with the probe hybridization solution and left to hybridize at 56°C for 12 hr. Following staining with DAPI for 30 min and subsequent PBS washing, samples were sectioned and observed under a fluorescence microscope.

### 2.8. Culture and Treatment of Human Aortic Endothelial Cells

Human aortic endothelial cells (HAECs) from the Type Culture Collection of the Chinese Academy of Sciences, Shanghai, China, were cultured in DMEM (Life Technologies, Grand Island, NY) and supplemented with 10% FBS (Sigma-Aldrich) and 1% penicillin–streptomycin (Solarbio, Beijing, China) at 37°C with 5% CO_2_. Shear stress was induced in HAECs following the methodology outlined in our previous study [33].

HAECs were exposed to AS-IV and precultured for 24 hr. AS-IV concentrations were set to 0, 50, 100, 150, and 200 *μ*mol/L [34].

### 2.9. Transfection of HAECs

Plasmids containing the miR-17-3p mimic and the corresponding negative control (NC) were procured from Sangon Biotech Co., Ltd. (Shanghai, China). Transfection of HAECs with these plasmids was achieved using the Lipofectamine™ 2000 reagent (Life Technologies, Carlsbad, CA, USA) at a multiplicity of infection of five. In the control group, HAECs were transfected with NC plasmids.

### 2.10. Dual-Luciferase Reporter Assay

In the dual-luciferase reporter assay, 1 *μ*g of wild-type-SDC1-pGL4 (Promega Corporation, USA) or mutant-SDC1-pGL4 (induced using QuickMutation™ Kit, Beyotime, China), along with 50 nmol of miR-17-3p or NC, and 150 ng of Renilla (RG062M, Beyotime, China), were transfected into 3 × 10^4^ cells using Lipofectamine® 2000. The treated cells were incubated at 37°C for 36 hr. Luciferase activity was assessed using a dual-luciferase reporter gene detection kit (Promega, USA).

### 2.11. Real-Time Quantitative Reverse Transcription PCR (RT-qPCR)

Cells or tissues were collected in 1.5-mL EP tubes and treated with 1 mL of TRIzol on ice. For every 1 mL of TRIzol used, 0.2 mL of chloroform was added. The samples were vigorously mixed for 15 s, incubated at 25°C for 3 min, and centrifuged at 12,000x *g* for 15 min at 4°C. The upper aqueous phase was transferred to a new tube, and RNA in the aqueous phase was precipitated with isopropanol. After centrifugation under the aforementioned conditions for 10 min, the supernatant was discarded, and the RNA pellet was washed with 75% ethanol. For every 1 mL of TRIzo1 used, at least 1 mL of 75% ethanol was added. The samples were centrifuged at 7,500x *g* for 5 min at 4°C. The RNA pellet was air-dried at 25°C for approximately 3–5 min, and 25 *µ*L of RNase-free water was added to dissolve the RNA. The PrimeScript RT Kit (#DRR037A, Takara) and miRNeasy Mini Kit (#217004, Qiagen) were used to synthesize cDNA from SDC1 mRNA and miR-17-3p, respectively. SYBR Green reagent (#A2203XL, Yaji, China) and TaqMan miRNA (KMD122014, KEMOBio, China) were used for quantitative polymerase chain reaction (qPCR). The expression of SDC1 mRNA and miR-17-3p was normalized to that of GAPDH and U6 using the 2^−*ΔΔ*Ct^ method. The PCR primer sequences are as follows:

  SDC1-F, 5′-ATGGCTCTGGGGATGACTCT-3′;
  SDC1-R, 5′-GCTGCCTTCGTCCTTCTTCT-3′;
  GAPDH-F, 5′-GCACCGTCAAGGCTGAGAAC-3′;
  GAPDH-R, 5′-TGGTGAAGACGCCAGTGGA-3′;
  miR-17-3p-F, 5′-ACACTCCAGCTGGGACTGCAGTGAAGGCAC-3′;
  miR-17-3p-R, 5′-TGGTGTCGTGGAGTCG-3′;
  U6-F, 5′-CTCGCTTCGGCAGCACATATACT-3′;
  U6-R, 5′-ACGCTTCACGAATTTGCGTGTC-3′.

### 2.12. Western Blotting

Following the removal of the culture medium and thorough washing of cells or tissues, total protein was isolated from 100 *μ*L of lysates prepared using 1 *μ*L of protease inhibitor and 1 *μ*L of PMSF. After centrifugation at 12,000x *g* for 15 min at 4°C, the supernatant was collected in a 1.5-mL EP tube. Subsequently, 2.5 *μ*L of the sample was diluted in 22.5 *μ*L of triple-distilled water to measure the concentration of extracted proteins using BCA. Proteins were extracted, separated on 8% SDS–polyacrylamide gel electrophoresis, and transferred onto PVDF membranes. The membranes were then incubated with a primary antibody (1 : 1,000, ab128936) overnight at 4°C. The following day, membranes were incubated with a secondary antibody (1 : 2,000, ab6721) for 2 hr at 37°C. Protein bands were visualized using an ECL kit (Amersham Biosciences, Piscataway, NJ) and the IPP6.0 software, and the relative expression of proteins was normalized to that of GAPDH (internal reference).

### 2.13. Immunofluorescence Staining

Aortic tissues were fixed in 4% paraformaldehyde/PBS (*w*/*v*), permeabilized with 0.5% of Triton X-100 (prepared in PBS) for 20 min, and blocked with normal goat serum (5% BSA) at 25°C for 30 min. After absorbing the excess blocking solution with absorbent paper, tissues were incubated with a sufficient amount of mouse monoclonal anti-chondroitin sulfate (CS) antibody (ab11570, 1 : 500, diluted with 5% BSA) in a wet box at 4°C overnight. The following day, tissues were incubated with FITC-conjugated secondary antibody (ab6717, 1 : 1,000) in a humid chamber at 20–37°C for 1 hr. From this step onward, all subsequent procedures were conducted in the dark. Nuclei were stained with DAPI for 5 min. Subsequently, tissues were mounted and imaged using a fluorescence microscope (pE 300 Lite, CoolLED, UK).

### 2.14. CCK-8 Assay

Cells (100 *μ*L) were seeded in 96-well plates at a concentration of 5 × 10^4^ mL. After incubation, 10 *μ*L of CCK-8 solution was added, and plates were gently placed on an orbital shaker for 1 min at 37°C to ensure uniform mixing. Subsequently, cells were incubated for 2 hr for dehydrogenation. Optical density values at 450 nm were measured using a microplate reader (HBS-1096A, Detie, China).

### 2.15. Flow Cytometry

Flow cytometry (BD FACSCalibur, Becton Dickinson, USA) was used to assess the apoptotic rate. Cells were incubated with 5 *μ*L of annexin V-FITC and PI (Sanjian Biological Technology Co., Ltd., Chongqing, China) for 15 min in the dark, followed by analysis using flow cytometry.

### 2.16. Statistical Analysis

Statistical analysis was conducted using GraphPad software (version 7.0). Data were presented as mean ± standard deviation. A *t*-test was employed for comparing means between two groups, and a one-way analysis of variance and Tukey's multiple comparison test were performed for comparing means among three or more groups. A *p* value of <0.05 was considered statistically significant.

## 3. Results

### 3.1. AS-IV Alleviates AAA

**Figure 1:**
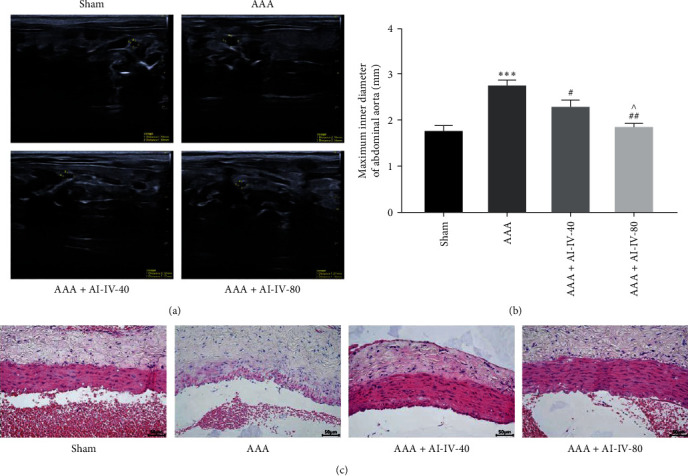
AS-IV alleviates AAA. Rats were categorized into four groups (*n* = 10): Sham, AAA, AAA + AS-IV-40, and AAA + AS-IV-40. AAA rat model was constructed using PPE, and the rats in the Sham group received heat-inactivated PPE. AS-IV was administered at doses of 40 mg/kg and 80 mg/kg via gavage. (a,b) Comparison of the aortic inner diameter of rats in each group. (c) The histopathological changes of the abdominal aorta in each group were analyzed using HE staining.  ^*∗∗∗*^*P* < 0.001 vs. Sham group; ^#^*P* < 0.05, ^##^*P* < 0.01 vs. AAA group; ^^^*P* < 0.05 vs. AAA + AI-IV group. AS-IV, astragaloside IV; and AAA, abdominal aortic aneurysm.

To assess the impact of AS-IV on AAA, rats in the AAA group were administered AS-IV at doses of 40 and 80 mg/kg through gavage. The results revealed a significant increase in the maximum aortic inner diameter in the AAA group, confirming successful modeling (*P* < 0.001, Figures 1(a) and 1(b)). Both AS-IV doses effectively inhibited aortic inner diameter (*P* < 0.05 and *P* < 0.01), with the 80 mg/kg dose demonstrating a more pronounced therapeutic effect (*P* < 0.05, Figures 1(a) and 1(b)). In addition, the Sham group exhibited a normal aorta. In the AAA group, the inner and outer aorta exhibited irregularities, incomplete structure, and degenerated elastic fibers. The aortic damage in the AAA + AS-IV-40 and AAA + AS-IV-80 groups was significantly reduced compared to the AAA group. In these groups, the aortic intima appeared smoother, and the middle layer of cells was denser. Notably, the AAA + AS-IV-80 group exhibited a lesser degree of damage (Figure 1(c)). These findings provide initial evidence that AS-IV possesses the potential to alleviate AAA and reduce tumor size.

### 3.2. AS-IV Upregulates SDC1 and Protects Glycocalyx Structure and Function in AAA Rats

**Figure 2:**
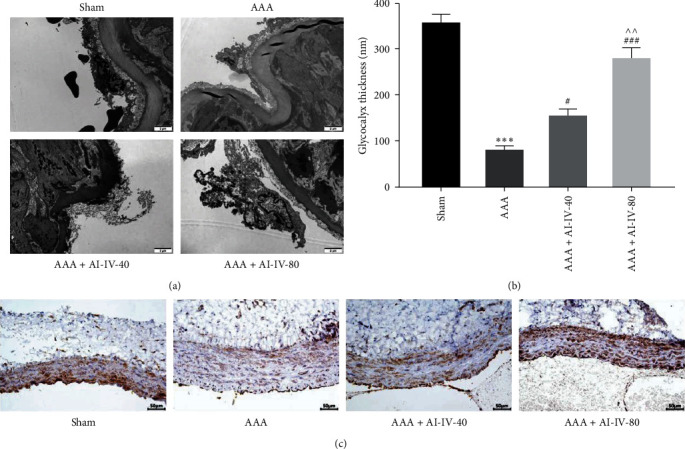
AS-IV upregulates SDC1 and protects glycocalyx structure and function in AAA rats. (a,b) Glycocalyx thickness in the abdominal aortic endothelial cells was observed using TEM. (c) Immunohistochemical staining was used to observe SDC1 expression, a key glycocalyx protein, in the abdominal aorta.  ^*∗∗∗*^*P* < 0.001 vs. Sham group; ^#^*P* < 0.05, ^###^*P* < 0.001 vs. AAA group; ^^*P* < 0.01 vs. AAA + AS-VI-40 group. AS-IV, astragaloside IV; AAA, abdominal aortic aneurysm; SDC1, syndecan-1; and TEM, transmission electron microscopy.

To investigate glycocalyx changes in AAA and the impact of AS-IV, the aortic endothelial glycocalyx was examined using transmission electron microscopy (TEM). The AAA model exhibited significantly reduced glycocalyx thickness (*P* < 0.001). AS-IV treatment effectively restored glycocalyx thickness and improved its structure (*P* < 0.05 and *P* < 0.001, Figures 2(a) and 2(b)). Further analysis of glycocalyx changes involved detecting the expression and localization of SDC1 protein through IHC. SDC1, a key glycocalyx protein, was prominently expressed on the endothelial cell membrane, and its downregulation leads to glycocalyx shedding. In the Sham group, SDC1 was abundant in aortic endothelial cells. The AAA group displayed a significant reduction in SDC1 protein levels. However, in the AAA + AS-IV-40 and AAA + AS-IV-80 groups, SDC1 expression rebounded notably, particularly in the AAA + AS-IV-80 group (Figure 2(c)). This preliminary evidence suggests that glycocalyx damage is implicated in the AAA progression in the PPE-induced rat model. AS-IV not only alleviates AAA but also enhances SDC1 expression, preserving glycocalyx structure and function.

### 3.3. AS-IV Inhibits miR-17-3p Expression in Endothelial Cells of AAA Rats

**Figure 3:**
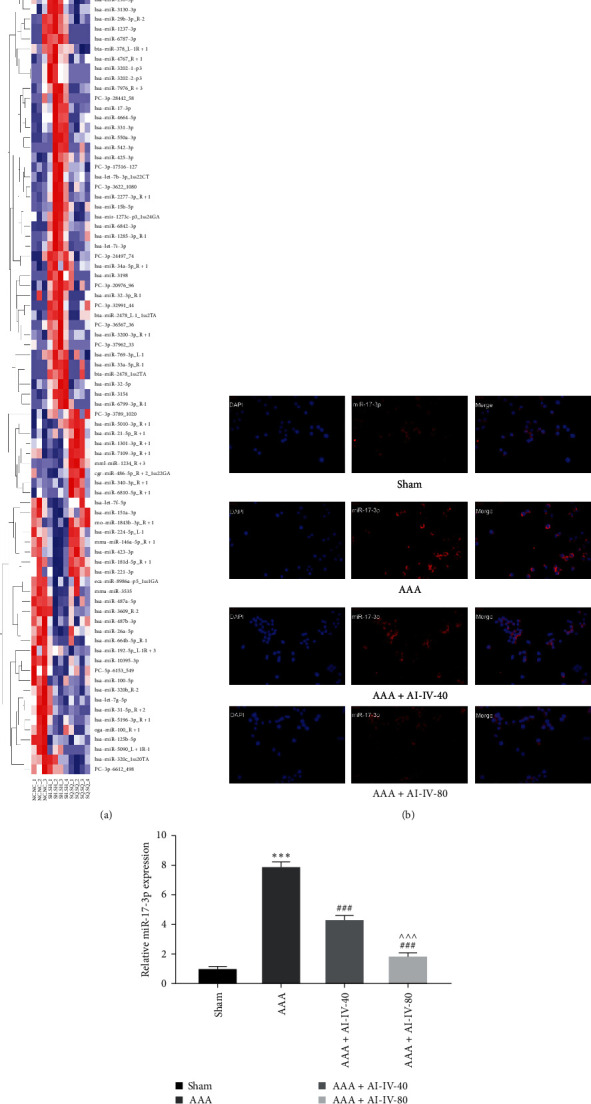
AS-IV inhibits miR-17-3p expression in endothelial cells of AAA rats. (a) The heat map shows different miRNAs in the peripheral blood of healthy individuals, untreated patients with AAA, and treated patients with AAA. (b) FISH was used to detect miR-17-3p expression in the abdominal aorta. (c) RT-qPCR was used to detect miR-17-3p expression in the abdominal aorta.  ^*∗∗∗*^*P* < 0.001 vs. Sham group; ^###^*P* < 0.001 vs. AAA group; ^^^*P* < 0.001 vs. AAA + AS-IV-40 group. AS-IV, astragaloside IV; AAA, abdominal aortic aneurysm; FISH, fluorescence in situ hybridization; and RT-qPCR, real-time quantitative reverse transcription PCR.

The miRNA expression profiles in blood samples were examined as previously described in our clinical study on patients with AAA [32]. Blood samples from healthy individuals, untreated patients with AAA, and treated patients with AAA identified 89 differentially expressed miRNAs (Figure 3(a)). Using TargetScan, miRNAs targeting SDC1 were predicted, revealing miR-17-3p as a key player associated with AAA. Sequencing results indicated elevated miR-17-3p levels in the peripheral blood of patients with AAA compared to healthy individuals, with a significant reduction posttreatment (Figure 3(a)). For preliminary verification, FISH and RT-qPCR assessed miR-17-3p expression in aortic endothelial cells of AAA rats. The results demonstrated low miR-17-3p expression in the Sham group and elevated levels in the AAA group (*P* < 0.001). Significantly, AAA + AS-IV-40 and AAA + AS-IV-80 groups exhibited reduced miR-17-3p expression, particularly in the AAA + AS-IV-80 group (*P* < 0.001, Figures 3(b) and 3(c)). These findings indicate a correlation between increased miR-17-3p expression in endothelial cells and AAA progression, with AS-IV effectively inhibiting miR-17-3p expression.

### 3.4. miR-17-3p Inhibits SDC1 Expression in HAECs

**Figure 4:**
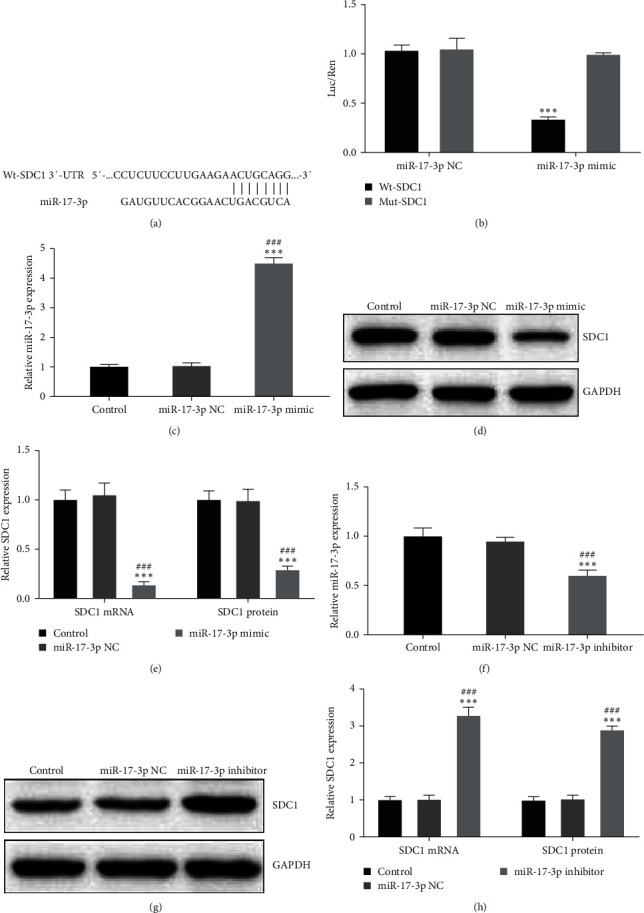
miR-17-3p inhibits SDC1 expression in HAECs. (a) The targeted binding sites of miR-17-3p and SDC1 mRNA. (b) The relationship between miR-17-3p and SDC1 was confirmed using the dual-luciferase report assay. (c) miR-17-3p overexpression by transfection. (d, e) Comparison of SDC1 mRNA and protein levels in HAECs in each group. (f) miR-17-3p silencing by transfection. (g, h) Comparison of SDC1 mRNA and protein levels in HAECs in each group.  ^*∗∗∗*^*P* < 0.001 vs. Mut-SDC1 group or Control group; ^###^*P* < 0.001 vs. miR-17-3p NC group. SDC1, syndecan-1 and HAECs, human aortic endothelial cells.

A dual-luciferase reporter assay was conducted to investigate the relationship between miR-17-3p and SDC1. The results confirmed the binding between miR-17-3p and SDC1 mRNA in HAECs (Figures 4(a) and 4(b)). Transfection with the miR-17-3p mimic significantly increased miR-17-3p expression in HAECs (*P* < 0.001, Figure 4(c)). Elevated miR-17-3p expression led to the inhibition of SDC1 mRNA and protein levels (*P* < 0.001, Figures 4(d) and 4(e)). Conversely, inhibiting miR-17-3p demonstrated a significant increase in SDC1 mRNA and protein levels (*P* < 0.001, Figure 4(f)–4(h)). These findings strongly indicate that miR-17-3p directly targets SDC1 in HAECs.

### 3.5. AS-IV Suppresses miR-17-3p Expression and Promotes SDC1 Expression in HAECs

**Figure 5:**
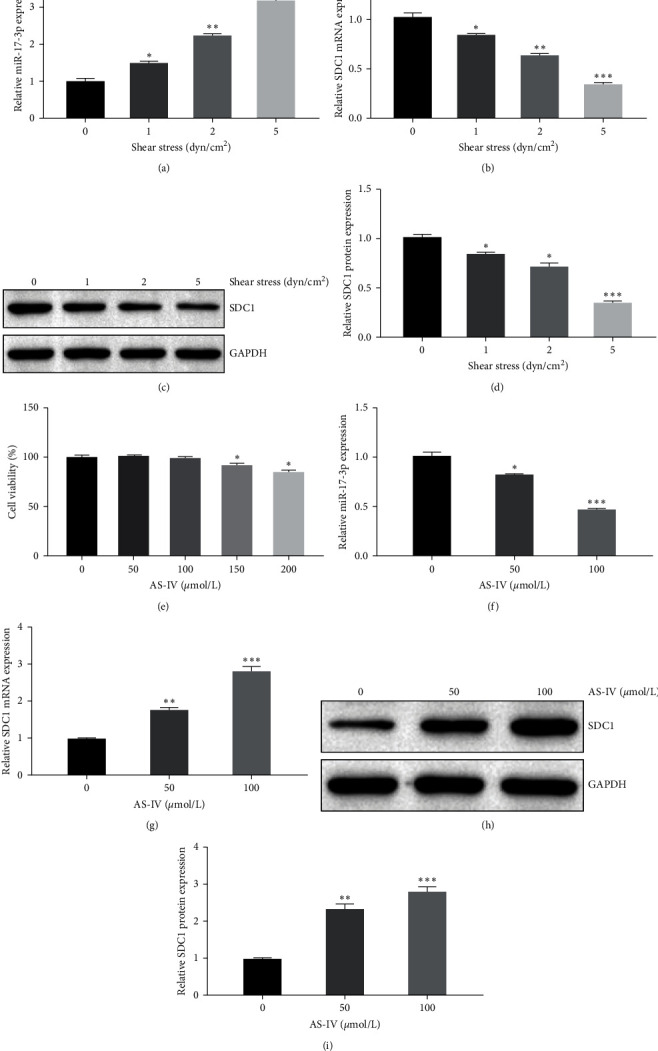
AS-IV inhibits miR-17-3p expression and promotes SDC1 expression in HAECs. (a) The impact of shear stress on miR-17-3p expression in HAECs. (b–d) The impact of shear stress on SDC1 mRNA and protein expression in HAECs. (e) The impact of different AS-IV concentrations on cell viability of HAECs. (f) The impact of AS-IV on miR-17-3p expression in HAECs. (g–i) The impact of AS-IV on SDC1 mRNA and protein expression in HAECs.  ^*∗*^*P* < 0.05,  ^*∗∗*^*P* < 0.01,  ^*∗∗∗*^*P* < 0.001 vs. 0 dyn/cm^2^ shear stress group or 0 *μ*mol/L AS-IV group. AS-IV, astragaloside IV; AAA, abdominal aortic aneurysm; SDC1, syndecan-1; and HAECs, human aortic endothelial cells.

In HAECs subjected to varying shear stress intensities (0, 1, 2, and 5 dyn/cm^2^), miR-17-3p expression exhibited a significant increase with escalating shear stress (*P* < 0.05, *P* < 0.01, and *P* < 0.001; Figure 5(a)). However, higher shear stress levels correlated with decreased SDC1 mRNA and protein expression in HAECs (*P* < 0.05, *P* < 0.01, and *P* < 0.001; Figure 5(b)–5(d)). These findings are consistent with in vivo experiments, indicating a parallel increase in miR-17-3p expression and a decrease in SDC1 expression in AAA.

To evaluate the impact of AS-IV on HAECs, cells were treated with 0, 50, 100, 150, and 200 *μ*mol/L of AS-IV for 24 hr. Concentrations of 150 and 200 *μ*mol/L induced cytotoxicity (*P* < 0.05), while ≤100 *μ*mol/L had no significant impact on cell viability (Figure 5(e)). Consequently, 50 and 100 *μ*mol/L concentrations were selected for subsequent experiments. AS-IV at these concentrations effectively suppressed miR-17-3p expression in HAECs, with the 100-*μ*mol/L dose exhibiting a significant inhibitory effect (*P* < 0.001, Figure 5(f)). Furthermore, both concentrations increased the SDC1 mRNA and protein expression in HAECs, with the 100 *μ*mol/L concentration demonstrating a more significant effect (*P* < 0.001, Figure 5(g)–5(i)). These findings suggest that AS-IV, within safe concentrations, can inhibit miR-17-3p expression while enhancing SDC1 mRNA and protein expression in HAECs.

### 3.6. AS-IV Mitigates Shear Stress–Induced Damage in HAECs via miR-17-3p Inhibition

**Figure 6:**
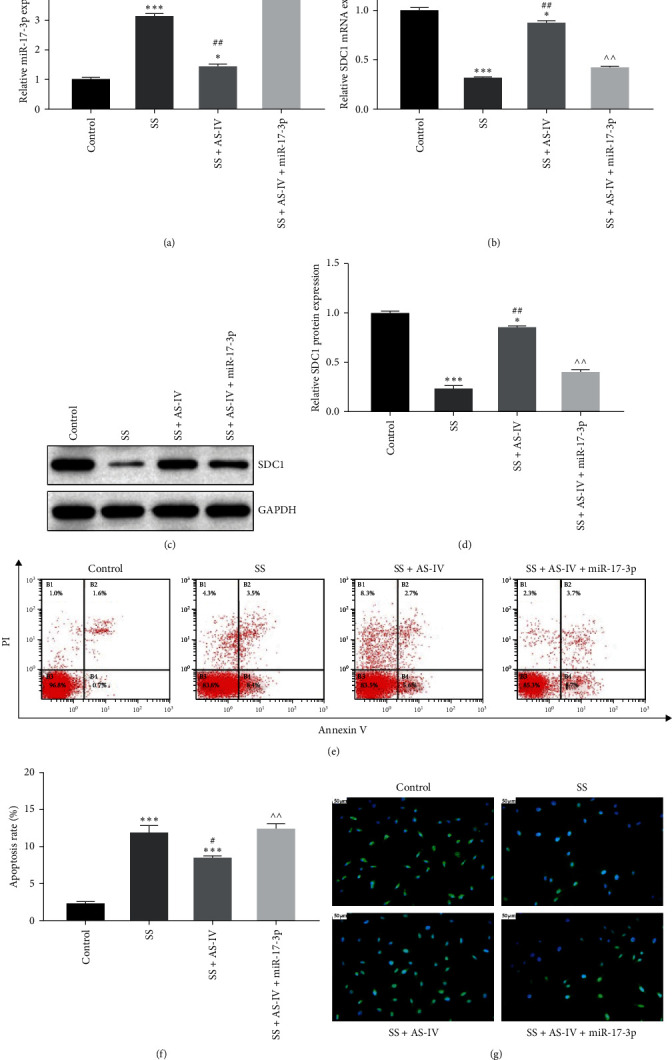
AS-IV mitigates shear stress-induced damage in HAECs via miR-17-3p inhibition. HAECs were divided into four groups: control, SS (5 dyn/cm^2^ shear stress), SS + AS-IV (100 *μ*mol/L), and SS + AS-IV + miR-17-3p. (a) Comparison of miR-17-3p expressions in each group. (b–d) Comparison of SDC1 mRNA and protein expressions in each group. (e,f) Comparison of cell apoptosis rates, including early and late apoptosis, in each group. Early apoptosis was observed in the B4 quadrant, and late apoptosis was observed in the B2 quadrant. (g) Immunofluorescence staining revealed the CS expression level to assess the glycocalyx damage.  ^*∗*^*P* < 0.05,  ^*∗∗∗*^*P* < 0.001 vs. Control group; ^#^*P* < 0.05, ^##^*P* < 0.01 vs. SS group; ^^^^*P* < 0.01, ^^^^^*P* < 0.001 vs. SS + AS-IV group. AS-IV, astragaloside IV; HAECs, human aortic endothelial cells; SS, shear stress; and CS, chondroitin sulfate.

To elucidate the role of miR-17-3p in the therapeutic effects of AS-IV on AAA, miR-17-3p overexpression was induced, and shear stress (5 dyn/cm^2^) was applied in HAECs. As observed previously, shear stress significantly upregulated miR-17-3p expression in HAECs (*P* < 0.001), while AS-IV countered this effect by inhibiting miR-17-3p expression under shear stress (*P* < 0.01, Figure 6(a)). In addition, AS-IV mitigated shear stress-induced declines in SDC1 mRNA and protein expression in HAECs (*P* < 0.01). Notably, miR-17-3p overexpression significantly blocked the enhancing effects of AS-IV on SDC1 mRNA and protein expression (*P* < 0.01, Figure 6(b)–6(d)). Shear stress induced apoptosis in HAECs (*P* < 0.001), and AS-IV attenuated apoptosis under shear stress (*P* < 0.05). Moreover, miR-17-3p overexpression significantly diminished the antiapoptotic effects of AS-IV, including early and late apoptosis (*P* < 0.01, Figures 6(e) and 6(f)). SDC1 protein, integral to the glycocalyx skeleton on HAEC surfaces, binds to CS, forming the basic structure of glycocalyx. Immunofluorescence staining revealed that shear stress reduced CS expression on HAEC surfaces and disrupted the glycocalyx structure. AS-IV mitigated glycocalyx shedding, while miR-17-3p overexpression countered the protective effects of AS-IV on the glycocalyx (Figure 6(g)). These results indicate that AS-IV, through the miR-17-3p–SDC1 axis, alleviates shear stress-induced apoptosis and glycocalyx damage in HAECs.

## 4. Discussion

Shear stress is a direct contributor to aortic endothelial cell damage in AAA [35, 36]. Under the influence of shear stress, the glycocalyx undergoes continuous shedding. Once the glycocalyx layer reaches a certain thinness, it adversely affects the survival and function of vascular endothelial cells [37, 38]. The present study sheds light on the profound impact of shear stress on the glycocalyx of aortic endothelial cells in AAA. Furthermore, it elucidates the mechanism through which AS-IV intervenes in AAA treatment.

AS-IV (C_14_H_68_O_14_), a cycloastragenol derivative, is a potent small-molecule compound extracted from *Astragalus membranaceus* rhizomes [39, 40]. Its recognized worldwide attention stems from its demonstrated antitumor activity and vascular-protective functions [41–43]. AS-IV has shown efficacy in alleviating vascular endothelial cell dysfunction induced by high-glucose treatment [44]. It also regulates the diastolic function of aortic endothelial cells by regulating eNOS expression [45]. Furthermore, AS-IV alleviates PM2.5-induced pulmonary toxicity by inhibiting the NLRP3 inflammasome [46]. While previous research has reported the protective effects of AS-IV on aortic endothelial cells, limited studies have explored its role in alleviating AAA [24], with only one study highlighting its inhibitory effect on inflammatory cell infiltration. Notably, the impact of AS-IV on AAA and shear stress-induced damage to aortic endothelial cells remains unclear. In this study, AAA was induced in rats through PPE injection, followed by AS-IV administration via gavage. The results revealed that the glycocalyx layer in the aorta of rats with AAA was notably thinner, accompanied by decreased SDC1 expression. AS-IV significantly reduced aortic inner diameter, alleviated vascular endothelial damage, and increased the aortic glycocalyx layer thickness and SDC1 expression. These findings establish the potential of AS-IV in alleviating AAA and protecting the glycocalyx of aortic endothelial cells.

miRNAs, conserved, short single-stranded RNA molecules, play a role in inhibiting protein expression by targeting the 3′-UTR of mRNAs at the posttranscriptional level, contributing to AAA development [47, 48]. In this study, sequencing of peripheral blood samples identified 89 differentially expressed miRNAs among healthy individuals, untreated patients with AAA, and treated patients with AAA. To screen miRNAs linked to AS-IV and SDC1, miRNAs targeting SDC1 were predicted, revealing miR-17-3p as a common candidate. miR-17-3p is known to regulate vascular endothelial cell function and influence AAA progression [27]. Sambri et al. [49] demonstrated that miR-17-3p modulates inflammation in vascular endothelial cells by targeting neuron-derived orphan receptor-1. Furthermore, miR-17-3p exhibits inhibitory effects on vascular endothelial cell proliferation and angiogenesis [50]. Conversely, it promotes endothelial progenitor cell differentiation to endothelial cells, facilitating AAA repair [51]. FISH analysis in this study revealed increased miR-17-3p expression in aortic endothelial cells of AAA rats, with AS-IV significantly inhibiting miR-17-3p expression. In addition, miR-17-3p hindered SDC1 protein expression in HAECs. These findings highlight the involvement of miR-17-3p in AAA development by targeting SDC1 to damage the glycocalyx and emphasize the potential of AS-IV in alleviating AAA by inhibiting miR-17-3p expression to preserve the glycocalyx.

To examine the impact of shear stress on the glycocalyx in AAA, HAECs underwent varying shear stress intensities. As shear stress reached ≤5 dyn/cm^2^, miR-17-3p expression increased, while SDC1 expression decreased with increasing shear stress intensity. AS-IV countered this trend by inhibiting miR-17-3p expression and boosting SDC1 mRNA and protein expression in HAECs. For insights into the role of miR-17-3p in the protective effects of AS-IV on the glycocalyx, HAECs were categorized into four groups: control, SS (shear stress), SS + AS-IV, and SS + AS-IV + miR-17-3p. AS-IV inhibited shear stress-induced miR-17-3p elevation, prevented shear stress-induced SDC1 mRNA and protein expression, and mitigated shear stress-induced apoptosis and glycocalyx damage. However, miR-17-3p overexpression blocked the positive impact of AS-IV on SDC1 expression, its glycocalyx-preserving effect, and its apoptotic inhibition. These findings suggest that AS-IV reduces shear stress-induced apoptosis and glycocalyx damage in HAECs through the miR-17-3p–SDC1 axis.

## 5. Conclusion

In summary, miR-17-3p emerges as a potential culprit in glycocalyx damage to aortic endothelial cells by targeting SDC1 expression, implicating its involvement in AAA. Meanwhile, AS-IV appears to counteract this process by upregulating SDC1 expression through miR-17-3p inhibition, thereby protecting the glycocalyx and alleviating AAA. This shows AS-IV as a promising avenue for AAA alleviation, warranting further investigation into its efficacy and safety. A deeper understanding of the mechanisms underlying AS-IV in miR-17-3p expression and its protective role in glycocalyx maintenance is crucial for comprehensive insights.

## Data Availability

The datasets used or analyzed during the current study are available from the corresponding author on reasonable request.
